# Hormonal contraception and mental health: a narrative review of screening, monitoring, and patient-centered care for family physicians

**DOI:** 10.3389/fpsyt.2026.1846370

**Published:** 2026-07-08

**Authors:** Abdul Rehman Zia Zaidi, Hiba Elhassan, Amina Mariam Syed, Noor Waseem, Anush Muhammed, Laila Fida, Sidrah Fathima, Ismat Taha, Gazal Tannous, Shazia Irshad, Baraa Alghalyini

**Affiliations:** 1 Department of Family and Community Medicine, College of Medicine, Alfaisal University, Riyadh, Saudi Arabia; 2 College of Medicine, Alfaisal University, Riyadh, Saudi Arabia; 3 Department of Obstetrics and Gynecology, Dallah Hospital Nakheel, Riyadh, Saudi Arabia

**Keywords:** anxiety, depression, family medicine, hormonal contraception, mental health, patient-centered care, women’s health

## Abstract

**Background:**

Hormonal contraceptives are widely used worldwide, yet the relationship between contraceptive use and mental health outcomes remains an area of active clinical inquiry, with mixed evidence regarding potential risks and benefits. Family physicians require clear guidance on screening, monitoring, and counseling.

**Objective:**

To provide a narrative review of current evidence on the association between hormonal contraceptive use and mental health outcomes, and to offer practical, expert-informed recommendations for family physicians.

**Methods:**

We conducted a narrative review of literature published between 2000 and 2025 in PubMed, Cochrane Library, EMBASE, and PsycINFO, examining studies on hormonal contraceptive methods and mental health outcomes (depression, anxiety, and mood disorders) in women of reproductive age. A narrative design was selected because of marked heterogeneity in study designs, formulations, populations, and outcome measures. Methodological quality was self-assessed using the Scale for the Assessment of Narrative Review Articles (SANRA), and the strength of the evidence underlying each conclusion is indicated throughout.

**Results:**

Evidence on hormonal contraception and mental health was mixed. Some studies reported associations with increased depression and anxiety; others reported neutral or even protective associations. Individual variability was notable, influenced by age, pre-existing psychiatric history, and formulation. Progestin-only methods and initiation during adolescence were more consistently associated with higher mood-related risks, whereas certain combined oral contraceptives, particularly those with anti-androgenic progestins, were associated with better outcomes or mood stabilization. Because most evidence is observational, these associations cannot establish causation and are susceptible to confounding by indication, reverse causation, and selection effects including the healthy-user phenomenon.

**Conclusion:**

Given this variability, clinicians should prioritize individualized risk assessment, informed counseling, and close monitoring. Tailoring contraceptive choices to each woman’s mental health history is likely to support both reproductive autonomy and emotional well-being. The screening, risk-stratification, and monitoring approaches proposed here represent expert-informed clinical guidance rather than formal practice standards, and their feasibility will vary across primary care settings. Future research should focus on longitudinal designs, standardized outcomes, and diverse populations.

## 1 Introduction

### 1.1 Global context of contraceptive use

Globally, approximately 65% of women of reproductive age who are married or in a union are reported to be using some form of contraception, with modern contraceptive methods accounting for nearly 59% in this group (1). However, modern contraceptive prevalence rates vary considerably by region, with the proportion of demand satisfied with modern methods ranging from over 80% in parts of Europe and Eastern Asia to approximately 56% in Sub-Saharan Africa (2, 3).

Contraception can be hormonal or non-hormonal. Hormonal contraception (HC) contains either progestin alone or a combination of progestin and estrogen. It is available in several forms, including pills (combined and progestin-only), skin patches, vaginal rings, implants, injectables, and a hormonal intrauterine device (IUD) (4). Other non-hormonal methods of contraception include a copper IUD, male and female condoms, female and male sterilization, lactational amenorrhea method, withdrawal, and fertility awareness-based methods (1). The prevalence of methods used varies widely by region. Female sterilization and male condoms are the two most common methods used worldwide, followed by IUDs and OCPs. In Eastern and South-Eastern Asia, an IUD is the most common contraceptive method (18.6%), followed closely by male condoms (17%) (2).

Reproductive autonomy is defined as the power to decide and control matters associated with contraceptive use, pregnancy, and childbearing (5). Access to preferred contraceptives is not only a major determinant of reproductive autonomy but also advances several human rights. These include the right to life and liberty, freedom of opinion, work, and education.

Access to contraception also has implications for mental health, as a recent comprehensive review in World Psychiatry emphasized that there is a bidirectional relationship between reproductive health and psychiatric well-being across the female life course (6).

### 1.2 Mental health in women of reproductive age

Mental health is a fundamental human right and a rising global health concern. WHO characterizes anxiety by excessive worry, fear, and related behavioral disturbances, often severe enough to cause substantial distress or functional impairment. Depression is defined by persistent low mood, feelings of sadness, irritability, and loss of pleasure or interest in activities. Both conditions can trigger severe morbidity, disability, reduced productivity, and economic burden (7). There is increasing literature exploring the relationship between HC use and mental health outcomes such as depression and anxiety among women of reproductive age. A large cohort study investigating the prevalence of diagnosed mood disorders in reproductive-age women using hormonal contraceptives reported a figure of approximately 3.2 to 3.7% (8). For context, this estimate is broadly comparable to the 12-month prevalence of mood disorders reported in the general population of reproductive-age women, which limits the inference that can be drawn from a single-group figure reported without a contemporaneous non-user comparison group (7).

Hormonal influences on mood, particularly during the menstrual cycle, pregnancy, and menopause, are well documented. The menstrual cycle is characterized by changes in levels of estradiol and progesterone. These fluctuations may contribute to an increased risk of anxiety among women. Approximately 45% of women with a history of generalized social anxiety reported an influence on their social anxiety and avoidance symptoms during their menstrual cycle. Healthy women reported exacerbations of physiological symptoms during the late luteal and early follicular phases of the menstrual cycle, when levels of estradiol and progesterone are low (9).

The biological plausibility of an association between exogenous sex steroids and mood rests on several proposed and partly overlapping mechanisms, although none has been definitively established in humans. Estradiol modulates serotonergic and dopaminergic neurotransmission and influences the expression of serotonin receptors and the serotonin transporter, pathways closely tied to affective regulation (10). Progesterone is metabolized to the neuroactive steroid allopregnanolone, a positive allosteric modulator of the gamma-aminobutyric acid type A (GABA-A) receptor; rapid shifts in allopregnanolone availability are thought to contribute to mood symptoms during the premenstrual and postpartum periods (11). Synthetic progestins differ from endogenous progesterone in their affinity for progesterone, androgen, and glucocorticoid receptors and in their capacity to generate GABAergic neuroactive metabolites, which may help explain why formulations appear to differ in their mood profiles (11). Finally, exogenous steroids may alter hypothalamic-pituitary-adrenal (HPA) axis stress reactivity, with both blunted and exaggerated cortisol responses described in users (10). These mechanisms are presented as candidate explanations rather than confirmed causal pathways.

Moreover, the rates of depression are more significant among pregnant women compared with non-pregnant women. Vulnerable populations, such as adolescent mothers, are more likely to develop depression, anxiety and suicidal ideation compared to non-pregnant peers. They are also significantly more likely to develop antenatal and postnatal depression compared to adult mothers (12). Women with a past medical history of depressive symptoms during pregnancy are at an elevated risk of postpartum depression (PPD). The rapid changes in endogenous estrogen and progesterone levels during delivery contribute to PPD in vulnerable women (13).

In addition, existing literature also corroborates the link between reproductive hormones during the menopausal transition and depressive symptoms. A systematic review with a meta-analysis established that women who undergo menopause at an older age and experience a longer reproductive period are at a lower risk of developing depression in later life (14).

### 1.3 The contraception and mental health debate

There is a growing body of literature examining the association between hormonal contraceptives (HC) and mental health outcomes. This literature is genuinely mixed: although several studies report associations with worse mood, others describe neutral or protective associations, and because the evidence base is predominantly observational it cannot, on its own, establish causation (15, 16). Both estrogen and progesterone influence neurochemistry and brain function, which have been proposed to contribute to the negative mood changes and depression reported by some women taking oral contraceptive pills (8).

A comprehensive review included 23 controlled studies published between 2014 and 2024 investigating the relationship between mood and hormonal contraceptive use. Three studies demonstrated a decrease in depressive symptoms with the use of HCs, three demonstrated no significant trend or association, and the remaining studies reported a positive correlation between HC use and depressive symptoms and mood disorders (17).

A widescale population-based cohort using UK Biobank data found that the use of OCs, particularly during the first two years, was associated with an increased risk of depression. Additionally, adolescents who used OCs may also be more susceptible to depression in the future (18). Because this study used a within-sibling comparison to reduce shared familial and genetic confounding, it offers stronger support for a causal interpretation than most observational reports, although residual confounding cannot be excluded (18). Another nationwide population-based cohort study from the Danish registry found that postpartum hormonal contraceptive initiation was associated with an immediate increase in the risk of depression. The association was greater when hormonal contraception was initiated earlier in the postpartum period, and this association was most pronounced for combined oral contraceptive pills (19). Although evidence shows that different types of HCs may have different effects on mood and depression risk, a lack of literature and research about women’s biology, or the mechanism of action of HCs on depression, makes it challenging to identify the specific factors that are most associated (17).

This gap in mechanistic understanding creates a clinical dilemma for healthcare providers, who must weigh the reproductive benefits of hormonal contraception against uncertain psychological risks in the absence of definitive causal evidence. A 2025 research review further highlighted that sampling biases, particularly the common practice of combining ‘never users’ and ‘former users’ in analyses, may be masking long-term associations between adolescent hormonal contraceptive use and depression risk (20). A companion editorial has likewise argued that prevalent-user designs, healthy-user effects, and misalignment of the analytic time-zero are recurrent threats to validity across this literature, and that observational findings should therefore be interpreted as associations rather than established causal effects (21).

### 1.4 Clinical relevance for family physicians

Family physicians are often the initial point of contact for reproductive health services, including contraception. They play a crucial role in sexual and reproductive healthcare. Research shows that quality contraceptive counseling improves patient satisfaction with their contraceptive method, whereas poor-quality care worsens medical mistrust and discourages patients from seeking future care (22).

The focus of this review on family physicians is deliberate. In most health systems, primary care clinicians provide the majority of contraceptive prescriptions and are frequently the first, and sometimes only, clinicians to whom women report mood symptoms (22). Unlike a single specialist consultation, the longitudinal relationship characteristic of family practice allows repeated assessment of mood over the months during which contraceptive-related symptoms are most likely to emerge, and positions the family physician to integrate reproductive and mental health care within one therapeutic relationship (6). Accordingly, this review is intended primarily as a practical synthesis for primary care, rather than as a specialist reproductive-psychiatry reference.

Physicians should recognize the potential impact of contraceptives on mental health and conduct a detailed discussion to determine their patient’s preferences. Any past medical history of premenstrual depression or depression related to previous contraception should be documented, as this is often overlooked and may be associated with poorer outcomes. Providers are therefore well placed to adopt what some have called a “mindful prescribing” approach to contraceptive counseling (17).

The CDC emphasizes the significance of evidence-based guidelines and promotes clinical judgments grounded in patient-centered care, safety, and efficacy. Physicians are encouraged to apply a tiered counseling approach, prioritizing the most effective methods while respecting the patient’s reproductive autonomy and individual preferences. This approach aims to standardize contraceptive care and decision-making across primary care settings (23).

### 1.5 Review objectives

This narrative review aims to: (1) synthesize current evidence on the relationship between hormonal contraceptive use and mental health outcomes, (2) identify factors that may influence individual risk, and (3) provide practical recommendations for family physicians regarding screening, monitoring, and patient-centered care. It is intended as a practical synthesis for family physicians and other primary care clinicians, and the screening and monitoring suggestions offered are framed throughout as proposed, expert-informed guidance rather than formal clinical guidelines.

## 2 Methods

### 2.1 Search strategy and study selection

This article is a narrative review. A narrative rather than a systematic design was chosen deliberately, because the relevant literature is highly heterogeneous in study design, contraceptive formulation, age group, psychiatric outcome, and follow-up duration, and a narrative synthesis permits integration of mechanistic, epidemiological, and clinical sources that a single quantitative protocol could not readily accommodate. No protocol was registered and no meta-analysis was performed; accordingly, the review is reported with reference to the Scale for the Assessment of Narrative Review Articles (SANRA) rather than to the PRISMA statement, which is intended for systematic reviews (24, 25).

We conducted a comprehensive narrative review of the existing literature to examine the relationship between hormonal contraceptive use and associated mental health outcomes, namely depression, anxiety, and mood disorders. Electronic databases including PubMed, Cochrane Library, EMBASE, and PsycINFO were searched for studies published between January 2000 and December 2025. Our search strategy incorporated multiple combinations and variations of terms including hormonal contraception, oral contraceptives, contraceptive use, mental health, depression, anxiety, mood disorders, and psychological distress, using Boolean operators to refine the results. Filters were applied to limit retrieval to English-language publications. The reference lists of selected studies were also reviewed to identify additional relevant publications.

To improve reproducibility, a representative PubMed search string is reported here: (“hormonal contraception” OR “oral contraceptive*” OR “combined oral contraceptive*” OR “progestin-only” OR “intrauterine device” OR “levonorgestrel” OR “depot medroxyprogesterone” OR “contraceptive implant”) AND (“depression” OR “anxiety” OR “mood” OR “mood disorder*” OR “affective” OR “mental health” OR “psychological distress” OR “suicid*”) AND (“women” OR “adolescent*” OR “reproductive age”). Equivalent strings, adapted to the controlled vocabulary of each database (MeSH in PubMed, Emtree in EMBASE), were applied in EMBASE, PsycINFO, and the Cochrane Library. The final search was run on 31 December 2025.

The quality of this narrative review was self-assessed using the SANRA instrument (24). The initial search yielded approximately 850 records. After removing duplicates and screening titles and abstracts, 120 full-text articles were assessed for eligibility. A total of 65 studies met the inclusion criteria and were included in this narrative synthesis. **Table 1** presents a selection of eleven representative studies chosen to illustrate the breadth of designs, populations, and findings synthesized in this review; they do not constitute the totality of included studies.

**Table 1:** Summary of key included studies on hormonal contraception and mental health outcomes.

Study	Design	Sample	HC type	Key finding	Effect size	Limitations and comments
Skovlund et al. (26)	Prospective cohort	1,061,997 Danish women aged 15-34	All HC types (COCs, POPs, patches, rings, IUDs, implants)	First antidepressant use and depression diagnosis increased with HC use	RR 1.23 (95% CI 1.22-1.25) for antidepressants	Large national registry; no baseline mental health assessment; cannot confirm clinical depression; potential confounding by indication
Anderl et al. (27)	Longitudinal cohort	1,236 women (Add Health)	OCs in adolescence	Adolescent OC users had 1.7x higher odds of adult depression	OR 1.7 for adult depression	Nationally representative; long follow-up; self-reported OC use; recall bias; no HC type distinction
Johansson et al. (18)	Population cohort	264,557 UK Biobank women	OCs	OC use associated with depression risk, highest in first 2 years; sibling analysis strengthens causal interpretation	HR 1.71 (95% CI 1.48-1.98) first 2 years	Large cohort; sibling design; retrospective recall; limited HC formulation data
Larsen et al. (19)	Registry cohort	>610,000 postpartum Danish women	All postpartum HC	Postpartum HC initiation associated with increased depression incidence	21 vs 14 per 1,000 person-years	Large registry; controlled for covariates; observational; cannot exclude confounding by indication
Doornweerd et al. (28)	Prospective longitudinal	178 Dutch girls aged 16	OCs	OC users showed stable mood trajectories comparable to non-users	No significant group differences	Standardized instruments; repeated measures; small sample; self-selected OC use
Cheslack-Postava et al. (29)	Cross-sectional (NHANES)	2,607 US women aged 25-34	OCs	Current OC users had lower prevalence of depression and anxiety	Lower odds of current depression	Nationally representative; validated instrument; cross-sectional; cannot determine temporality
Kraft et al. (15)	Systematic review and meta-analysis	Multiple studies	All OCs	Mixed results across studies; high heterogeneity	Variable across studies	Comprehensive search; meta-analytic pooling; high between-study heterogeneity
McCloskey et al. (30)	Systematic review	Women with psychiatric disorders	Various HC	Minimal or positive mood effects; reduced suicide attempts	OR 0.37 for suicide attempts	Clinically relevant focus; variable study quality; selection bias likely
Toffol et al. (16)	Population survey	Finnish women	Current OC users	Current OC users had lower depressive symptom scores	Lower BDI-13 scores	Population-based; cross-sectional mood assessment; potential healthy-user bias
Burger et al. (31)	Systematic review	Multiple studies	LNG-IUD	Inconsistent evidence for mood effects of LNG-IUD	Variable findings	Focused review; variable study quality; heterogeneous methods
Mengelkoch et al. (17)	Comprehensive review	23 controlled studies (2014-2024)	All HC	Age and psychiatric history are key risk factors for HC-related mood changes	Variable across subgroups	Comprehensive and current; narrative synthesis limits quantitative conclusions
Howard et al. (6)	Expert review	Multiple populations	All reproductive HC	Bidirectional relationship between reproductive health and psychiatric outcomes	N/A (narrative)	Expert synthesis, not meta-analysis

HC, hormonal contraception; OC, oral contraceptive; LNG-IUD, levonorgestrel-releasing intrauterine device; RR, relative risk; OR, odds ratio; HR, hazard ratio; CI, confidence interval; BDI, Beck Depression Inventory.

**Figure 1:**
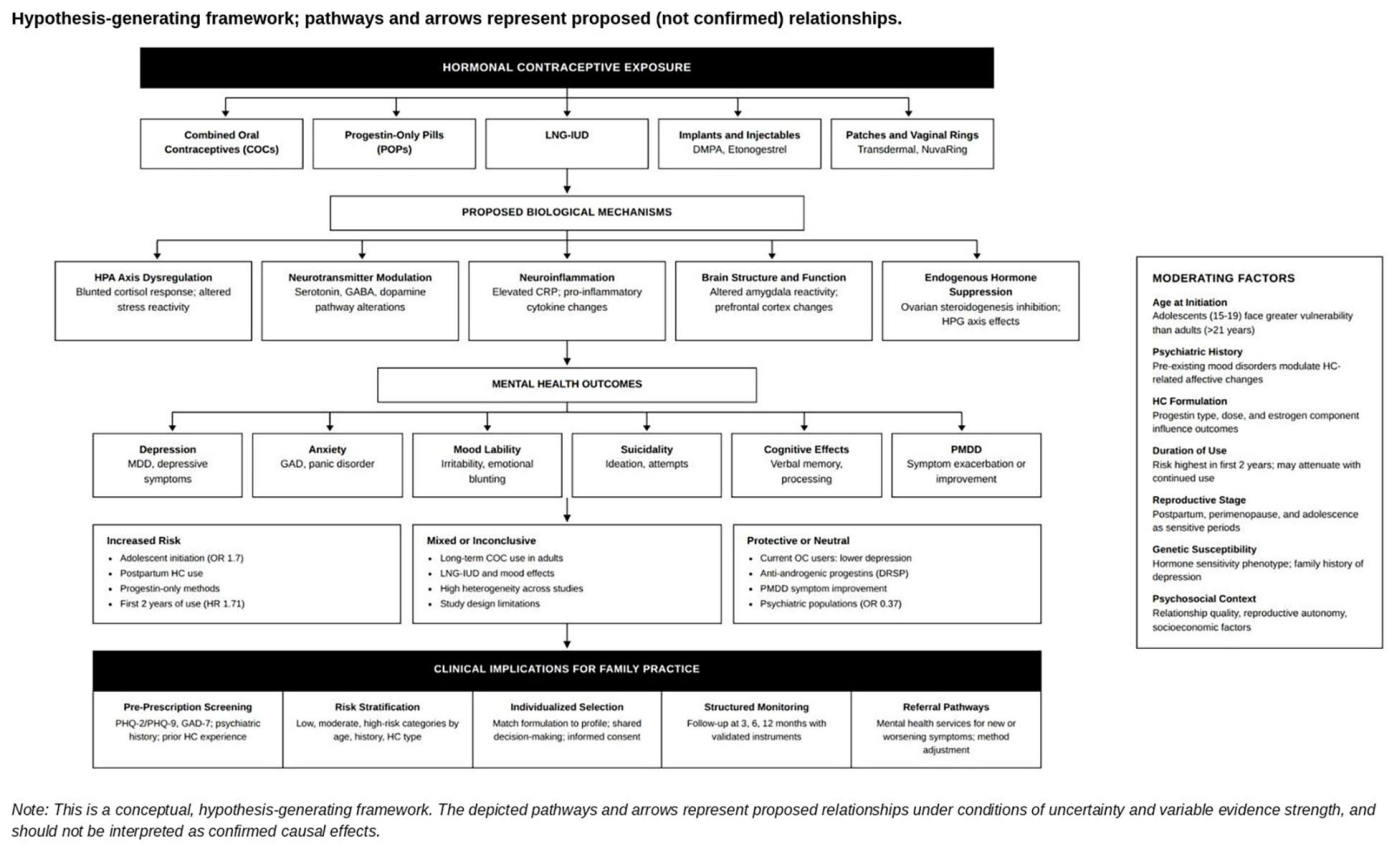
Conceptual framework illustrating the relationship between hormonal contraceptive exposure and mental health outcomes. The framework depicts categories of hormonal contraception, proposed biological mechanisms through which exogenous hormones may influence mood and cognition, the range of documented mental health outcomes, the direction of evidence (increased risk, mixed, or protective/neutral), and moderating factors that may influence individual susceptibility, together with clinical implications for family practice. This is a conceptual, hypothesis-generating framework; the depicted pathways and arrows represent proposed relationships under conditions of uncertainty and variable evidence strength, and should not be interpreted as confirmed causal effects. COCs, combined oral contraceptives; POPs, progestin-only pills; LNG-IUD, levonorgestrelreleasing intrauterine device; DMPA, depot medroxyprogesterone acetate; HPA, hypothalamic-pituitary-adrenal; HPG, hypothalamic-pituitarygonadal; GABA, gamma-aminobutyric acid; MDD, major depressive disorder; GAD, generalized anxiety disorder; PMDD, premenstrual dysphoric disorder; DRSP, drospirenone; PHQ, Patient Health Questionnaire; GAD-7, Generalized Anxiety Disorder 7-item scale.

The flow of records through identification, screening, eligibility assessment, and inclusion is summarized in **Figure 1**. Because this is a narrative review, the screening counts reflect the authors’ record of the selection process rather than a registered systematic-search audit trail.

### 2.2 Inclusion and exclusion criteria

#### 2.2.1 Inclusion criteria

Studies were eligible if they examined mental health outcomes, including mood changes, depression, or anxiety, in women using hormonal contraceptives, with a focus on women aged 15 to 45 years. Only peer-reviewed articles published in English within the period 2000 to 2025 were considered. Eligible designs included observational studies, clinical trials, and systematic reviews, in order to balance quality and relevance.

#### 2.2.2 Exclusion criteria

Studies were excluded if they were editorials, commentaries, conference abstracts, or involved non-human subjects. Studies focusing exclusively on non-hormonal contraception, those conducted in populations outside the reproductive age range, and studies lacking mental health outcome measures were also excluded.

### 2.3 Study types reviewed and selection criteria

To provide a comprehensive overview, we incorporated a range of study designs, including population-based cohort studies, cross-sectional surveys, randomized controlled trials, case-control studies, and systematic reviews. Both short-term and long-term studies were considered to capture the full spectrum of potential effects. Two reviewers independently screened the titles and abstracts of all identified studies to assess eligibility. Full-text articles of potentially relevant studies were then reviewed in detail. Data were extracted using standardized forms to maintain consistency and accuracy.

For each included study, we extracted the following data: first author and year of publication, study design, sample size and population characteristics, type of hormonal contraceptive evaluated, primary mental health outcome measures, key findings including effect sizes and confidence intervals where reported, and principal methodological limitations. Studies were not assigned formal quality scores, as a formal risk-of-bias assessment is not a standard requirement for narrative reviews; however, the methodological strengths and limitations of individual studies were considered during evidence synthesis and are noted in **Table 1** (24). The selection of studies for **Table 1** was guided by their relevance to the primary review objectives, the quality of their methodology, and their contribution to understanding the relationship between hormonal contraception and mental health outcomes.

### 2.4 Evidence appraisal and synthesis

Because narrative reviews do not employ a formal risk-of-bias instrument, we adopted a structured, transparent approach to appraising and weighting the evidence, and we report it here in response to reviewer concerns about critical appraisal and evidence quality.

First, the methodological quality of the present review was self-assessed against the six SANRA items, each scored from 0 (low standard) to 2 (high standard) for a maximum of 12 (24): (1) justification of the article’s importance for the readership; (2) statement of concrete aims or research questions; (3) description of the literature search; (4) referencing; (5) scientific reasoning, including balanced inclusion of evidence; and (6) appropriate presentation of data. After the present revisions, the authors self-assessed the review at 11 of 12, with one point withheld on item 3 because a narrative search does not provide the fully reproducible, audited search log expected of a systematic review.

Second, individual studies were appraised descriptively according to design and were assigned a qualitative strength-of-evidence label used throughout the Results and Discussion. Large prospective or registry-based cohort studies, randomized controlled trials, and quantitative meta-analyses were treated as stronger evidence; cross-sectional surveys, small pilot studies, and single case reports were treated as weaker, hypothesis-generating evidence. This labelling is intended to help the reader distinguish, for example, a nationwide registry cohort of more than one million women (26) from a two-patient case series (32).

Third, where findings conflicted, greater interpretive weight was given to larger, longitudinal, and confounding-adjusted studies, and to designs that reduce shared confounding (such as within-sibling comparisons), while inconsistencies were reported explicitly rather than resolved by selective emphasis (18, 19). We did not apply a formal GRADE assessment, which is designed for systematic reviews of comparable interventions; we note, however, that the certainty of the overall evidence base is low to moderate, and that this limited certainty is the reason the clinical recommendations in this review are framed as proposed guidance rather than as practice standards (33).

Finally, we acknowledge the inherent limitations of the narrative format: the absence of a registered protocol and a formal risk-of-bias tool, the potential for selection bias in the studies cited, and the dependence of the synthesis on the authors’ judgment. These limitations are discussed further in Section 4.8.

## 3 Results/evidence synthesis

### 3.1 Overview of current evidence

The literature reviewed included a variety of observational cohort studies, several systematic reviews, meta-analyses, and case-control studies. Most articles were published within the last 10 years, with a notable increase over the past 5 years, reflecting growing research interest in the intersection of contraception and mental health.

Synthesized analytically rather than study-by-study, the evidence shows a consistent pattern of inconsistency: the direction and magnitude of any association between hormonal contraception and mood depend heavily on study design, age group, and contraceptive formulation. The balance of larger studies suggests an association between hormonal contraceptive use and mental health outcomes, most notably depression and anxiety, but this association is neither uniform nor, on current evidence, demonstrably causal. Women with baseline depression have shown a more prominent increase in depressive symptoms in the first two years of taking hormonal contraception compared to women without baseline symptoms. There is, however, variability across demographics, particularly age: some studies indicate that women older than 30 experience more depressive and anxious symptoms, while others report that adolescents experience more severe symptoms. Women with lifetime exposure to hormonal contraception have been reported to show higher rates of anxiety and depression compared to never users, although this association is subject to confounding (27). The characteristics, designs, sample sizes, and principal findings of the key studies are summarized in **Table 1**.

Conversely, one study reported that women on oral contraception experienced fewer anxious and paranoid thoughts than during their normal menstrual cycle, and indicated that women on oral contraceptives had a lower prevalence of depression and anxiety compared to never-users; it also noted that women who used contraceptives throughout their lives had a lower risk of developing Alzheimer’s disease (15).

The strength of the evidence is variable, and this variability is central to its interpretation. Some studies had rigorous designs and adequate sample sizes, while others were limited by very short follow-up periods, lack of blinding, or non-standardized outcome definitions (34). In the synthesis that follows, we therefore give greater interpretive weight to large prospective and registry cohorts and to meta-analyses than to cross-sectional surveys, small pilot studies, and single case reports. Many studies relied on self-reported symptoms through questionnaires or surveys, which can introduce reporting bias and subjectivity (35). Studies also showed inconsistency in the hormonal formulations examined, with some failing to specify the type or dosage of contraceptive used, complicating cross-study comparison. Many studies lacked control over key variables such as socioeconomic status, pre-existing depression or anxiety, or concurrent medication use (36). Future research should adopt a more standardized protocol with longer follow-up, blinding where feasible, standardized outcome definitions, and more representative populations.

A 2025 commentary in World Psychiatry highlighted that the risk of depression associated with hormonal intrauterine systems appears to be hormone-dose-dependent, which the author interpreted as offering some support for a causal interpretation (37).

### 3.2 Effects on depression

#### 3.2.1 No overall correlation between oral contraceptive pills and depression

Most of the included analytic studies indicated no significant relationship between the use of oral contraceptive pills (OCP) and depression or symptoms of mental illness. Cross-sectional and longitudinal studies were not consistent in finding that hormonal contraceptive use is associated with an elevated risk of depression. For instance, O’Connell, Davis and Kerns (2007) found that although 48.6% of teenagers using OCPs had mild depression scores, this was not significantly different from non-users, which may indicate naturally elevated depressive symptoms among teenagers. Similarly, a report by Robinson et al. (38) indicated that mood shifts could be more closely associated with internal psychological expectations than with the pharmacological action of the drug.

#### 3.2.2 Protective or stabilizing associations of oral contraceptive pills

A small number of studies indicated that OCPs could have a protective or stabilizing influence on mood. Paoletti et al. (39) found that healthy women taking OCPs reported, in their third month of use, significantly fewer episodes of anxiety, phobias, and paranoid thoughts compared to naturally cycling women and to a control group. Likewise, Cheslack-Postava et al. (29) reported that women who had used OCPs in the past year had lower rates of depression and anxiety disorders than past or never users. Kim et al. (40) proposed that depressed women who use hormonal contraception may be less prone to Alzheimer’s disease, with potential long-term implications for cognitive health. Svendal et al. (41) noted that both positive and negative effects could occur depending on the specific pill used.

#### 3.2.3 Association between oral contraceptive pills and depression in young and teenage users

Although overall findings in adults are typically null, a number of large cohort studies have reported an association between OCP use and symptoms of depression among adolescents. Skovlund et al. (26) performed a large population study in Denmark and found that hormonal contraceptive use was associated with an elevated risk of antidepressant use, especially among adolescents; teenagers using the combined oral contraceptive pill were about 80% more likely to use antidepressants, and those using the progestin-only pill were more than twice as likely (overall relative risk for first antidepressant use 1.23, 95% CI 1.22 to 1.25). Likewise, Anderl, Li and Chen (27) reported that adolescents who had used OCPs were approximately 71% more likely (odds ratio approximately 1.7) to experience depression later in life, and that even two years after starting OCPs users had a 5% higher risk of depression than never users. A Swedish cohort indicated that girls aged 12 to 14 using non-oral progestin-only contraceptives were more than four times as likely to use mental health medications than peers who did not; this association was not observed in individuals older than 21.

#### 3.2.4 Mood effects associated with age and specific symptoms

Other research emphasizes developmental timing and specific depressive symptoms rather than syndromal depression. Using the Dutch-modified Reynolds Adolescent Depression Scale (RADS-2), one study reported that OCP-using adolescents had stable symptom patterns, whereas never-users developed an increase in symptoms during late adolescence (42). In another large sample, 16-year-old adolescent females using OCPs reported more indicators of depression, such as increased crying, hypersomnia, and appetite disturbance, but not an increase in core symptoms such as sadness or loss of pleasure (40). This suggests that OCP use might change the profile of depressive symptoms without necessarily producing a full clinical disorder, particularly during developmentally sensitive periods such as mid-adolescence.

#### 3.2.5 Psychological, social, and methodological considerations

Several studies have raised questions about whether psychosocial factors, rather than the pharmacological action of OCPs, may contribute to the mood shifts that have been observed. Cultural attitudes toward menstruation, sexuality, and contraception can influence how mood changes are interpreted and reported. Duke, Sibbritt and Young (43) reported that women who used OCPs for non-contraceptive reasons had higher odds of developing depression than women who used them for contraception (OR 1.32), with the association declining over longer duration of use, possibly reflecting psychological adaptation. Robinson et al. (38) reported that women given placebo pills complained of emotional side effects as often as those given active hormonal preparations, consistent with a nocebo effect. Where reproductive autonomy is limited, contraceptive use may be associated with stress or secrecy and may affect psychological well-being; effective counseling should therefore be culturally sensitive and attentive to the patient’s social environment and beliefs (44). Methodological limitations, including the scarcity of placebo-controlled trials and reliance on self-reported outcomes, remain (45, 46).

*Summary of evidence (depression).* Across designs, the evidence is mixed and design-dependent. The clearest signal of an adverse association comes from large registry and prospective cohort studies in adolescents, where reported estimates range from roughly a 5% relative increase to more than a doubling of risk (for example, hazard ratio 1.71, 95% CI 1.48 to 1.98, in the first two years of use in UK Biobank, and an approximately 80% higher likelihood of antidepressant use among teenage combined-pill users in Denmark) (18, 26). In adults, by contrast, cross-sectional and several registry analyses report null or even protective associations, including lower depression prevalence among current users and adjusted odds ratios below 1 for several combined formulations (29, 47). This divergence is consistent both with genuine age-related susceptibility and with methodological artefacts such as the healthy-user effect, and should not be interpreted as established causation.

### 3.3 Effects on anxiety

#### 3.3.1 General anxiety outcomes

A nationally representative sample of U.S. women aged 20 to 39 years showed that current use of oral contraceptives was associated with a reduced prevalence of anxiety-related disorders in the past year; women reporting OC use were less likely to meet diagnostic criteria for generalized anxiety disorder (GAD) and panic disorder (PD), with the exception of subthreshold major depressive disorder (MDD).

A population-based study in Finland found that current OC users had slightly lower scores on the Beck Depression Inventory-13, suggesting better mood, and that current OCP use was associated with a reduced occurrence of anxiety-related symptoms, including GAD and PD (16). A large U.S. study using NHANES data from 2005 to 2012 reported a significantly lower prevalence of major depression in current OCP users (4.6%) compared with previous (11.4%) and never users (10%). Although this study focused primarily on depression, it suggests that hormonal contraceptive use may be associated with improved mood outcomes; the prevalence of major depression was higher among women who were Black or Hispanic, widowed, divorced, or separated, of low income, smokers, antidepressant users, or who had a history of cancer or thyroid disease.

#### 3.3.2 Specific anxiety disorders

Panic disorder: A clinical report suggested a possible association between panic disorder and triphasic oral contraceptives, describing two women who developed classical panic symptoms months after initiating triphasic OCs, with resolution after discontinuation. This is a two-patient case series and constitutes weak, hypothesis-generating evidence only; more studies are needed, as the data on hormonal contraception and panic disorder are limited.

Generalized anxiety disorder: A nationally representative sample of U.S. women aged 20 to 39 years found that current OC users had a lower prevalence of all assessed mental health disorders except subthreshold MDD, including a reduced association with subthreshold GAD, suggesting a possible protective association.

Social anxiety: A case report by Roi and Conrad described a 34-year-old woman with social anxiety, panic disorder, premenstrual dysphoric disorder, and depression who experienced significant improvement in mood and anxiety, reduced suicidal thoughts, and better social functioning after starting a combined oral contraceptive (norgestimate-ethinyl estradiol), suggesting that combined oral contraceptives could relieve social anxiety symptoms in some women with hormone-related mood changes (32). As a single case report, this finding is illustrative rather than generalizable.

*Summary of evidence (anxiety).* The anxiety literature is smaller and methodologically weaker than the depression literature, resting substantially on cross-sectional surveys and isolated case reports rather than prospective cohorts. Within these limits, the available data more often suggest a neutral or modestly protective association between current oral contraceptive use and anxiety symptoms in adults, but the cross-sectional designs cannot establish temporality and are particularly vulnerable to the healthy-user effect. No firm conclusion about a causal effect on anxiety can currently be drawn.

### 3.4 Method-specific considerations

#### 3.4.1 Combined oral contraceptives (COCs)

When considering method-specific effects, it is important to differentiate between monophasic and multiphasic combined oral contraceptives (COCs). Monophasic COCs deliver a consistent dose of estrogen and progestin, whereas multiphasic COCs deliver varying doses across the menstrual cycle. One study found that monophasic COCs had a stronger favorable effect in panic disorder than multiphasic COCs (29). A low-dose monophasic COC (Minulet) was associated with significant mood improvements and reductions in depression, anxiety, and mood lability in women. The type of progestin in COCs may also influence mood: anti-androgenic progestins such as drospirenone have been associated with fewer adverse mood effects and are FDA-approved for treating premenstrual dysphoric disorder (48). However, data on androgenic progestins such as levonorgestrel (LNG) are inconclusive; some studies suggest that continuous LNG/ethinyl estradiol (EE) may reduce symptoms of PMDD and PMS including anxiety and mood lability, although these findings are inconsistent (49).

#### 3.4.2 Progestin-only methods

Progestin-only methods, including pills, implants, and injections, act by thickening cervical mucus, thinning the endometrium, and suppressing ovulation. The absence of estrogen in these methods could contribute to mood changes, as estrogen has known effects on neurotransmitter systems involved in mood regulation (50).

Some research suggests that progestin-only methods may be associated with an increased risk of depressive symptoms. A small pilot study found that women using progestin-only pills had significantly higher depression scores than those using combined oral contraceptives or non-users (51). This single pilot study, with a small sample and a surrogate biomarker outcome, constitutes weak evidence. Indeed, a review by McCloskey et al. (30) noted that some studies report higher rates of depression with progestin-only contraceptives while others report a protective association, yielding contradictory conclusions across the literature (30). The claim that progestin-only methods are more consistently associated with adverse mood outcomes should therefore be read as a tentative pattern across heterogeneous studies rather than as a well-established, high-certainty finding.

#### 3.4.3 Long-acting reversible contraceptives (LARCs)

LARCs, such as hormonal intrauterine devices (IUDs) and subdermal implants, provide effective contraception with minimal user intervention. The levonorgestrel IUD releases a small amount of progestin locally within the uterus. While some studies report improved quality of life and sexual functioning with hormonal IUDs, the evidence regarding mental health is mixed. A systematic review found inconsistent data on the mental health effects of levonorgestrel IUDs, with some studies indicating increased depressive symptoms and others showing no significant effect; the included studies varied in methodology, demographics, and quality, underscoring the need for more rigorous research (31).

Compared with other methods, LARCs may carry a lower risk of mood-related side effects owing to localized hormone release, although individual responses vary and some users may experience mood disturbance (50). Because LARCs act over 3 to 10 years, any mood-related effects may be subtle and develop gradually; early removal due to mental health concerns has been reported, highlighting the importance of monitoring and individualized counseling (52).

*Summary of evidence (method-specific effects).* Formulation appears to matter, but the supporting evidence is uneven. The most consistent signal is the favorable mood profile of combined oral contraceptives containing anti-androgenic progestins such as drospirenone, supported by their regulatory approval for premenstrual dysphoric disorder. Evidence that progestin-only methods carry a higher mood-related risk is weaker and inconsistent, resting in part on a single small pilot study, and evidence for levonorgestrel intrauterine systems is mixed. These differences should inform, but not dictate, individualized method selection.

### 3.5 Special populations

#### 3.5.1 Adolescents and young adults

Findings within adolescents merit particular attention, both because adolescence is a period of pronounced hormonal change and because many mental health conditions first emerge during this period. Doornweerd et al. (28) followed 178 girls aged 13 to 24 years using the standardized RADS-2 and SCARED instruments, with anxiety and depression measured on nine occasions. Never-users showed an increase in depression and anxiety symptoms in late adolescence, whereas symptom levels among OC users remained relatively stable across the trial; these differences persisted after adjustment for romantic relationships, sexual activity, education, and substance use, and did not appear to depend on the age at OC initiation. These results suggest that OC use might help stabilize mood during adolescence, although whether this confers long-term benefit or harm remains undetermined.

Conversely, Kraft et al. (15) conducted a systematic review identifying studies that reported a significant association between OC use and depression. For example, Anderl, Li and Chen (27) found that women who used OCs during adolescence were more likely to experience major depression as adults, and Lundin et al. (34) observed a minor increased risk of depression in adolescents using progestin-only contraceptives. These contradictory findings indicate that more focused studies are required to draw clearer conclusions.

#### 3.5.2 Women with pre-existing mental health conditions

A 2020 systematic review on contraception for women with psychiatric disorders found that most such women opt for combined oral contraceptives. Contraceptive provision in this group is important, as approximately 45% of pregnancies in the United States are unplanned, with higher rates among women with mental illness. Although LARCs are associated with fewer psychological side effects and lower failure rates, only about 14% of these women choose LARCs. Notably, a review of several double-blind, placebo-controlled studies found that hormonal contraceptive use in women with psychiatric disorders had minimal or even positive effects on mood; women using combined oral contraceptives had lower mean depressive symptom levels than non-users and were significantly less likely to report a past-year suicide attempt (odds ratio 0.37). This review urges greater consideration of mental health before prescribing contraceptives (30).

### 3.6 Hormonal withdrawal effects

Mental health problems associated with endogenous hormonal change appear most commonly during phases of hormonal withdrawal, such as the premenstrual or postpartum period or menopause (53). Similarly, withdrawal from hormone replacement therapy has been associated with negative mood changes (54).

Noachtar, Frokjaer and Pletzer (55) investigated whether the hormonal withdrawal during the 7-day break, or “pill pause,” during combined oral contraceptive (COC) use correlates with mental health symptoms. The study involved 181 participants in three groups: 61 women using androgenic COCs, 60 using anti-androgenic COCs, and 60 non-users with a natural menstrual cycle. Each participant was tested twice, once during the active pill or luteal phase and once during the pill pause or menses.

The findings revealed a significant mood deterioration in COC users during the pill pause, regardless of formulation, comparable in magnitude to the fluctuations experienced during the menstrual cycle by naturally cycling women. The authors concluded that withdrawal from contraceptive steroids during the pill pause is associated with mental health symptoms akin to those experienced during natural menstrual withdrawal, raising questions about the value of the pill pause from a mental health perspective (55).

These observations suggest that contraceptive regimens using continuous hormonal release could help reduce withdrawal-related symptoms. This is supported by a Cochrane review reporting that prolonged hormone use with short or no hormone-free intervals led to fewer menstrual-related symptoms, including mood symptoms, and that women using ethinylestradiol/norgestimate continuously did not experience the depressive symptoms reported with cyclic use (56).

## 4 Discussion

### 4.1 Synthesis of current evidence

**Figure 2:**
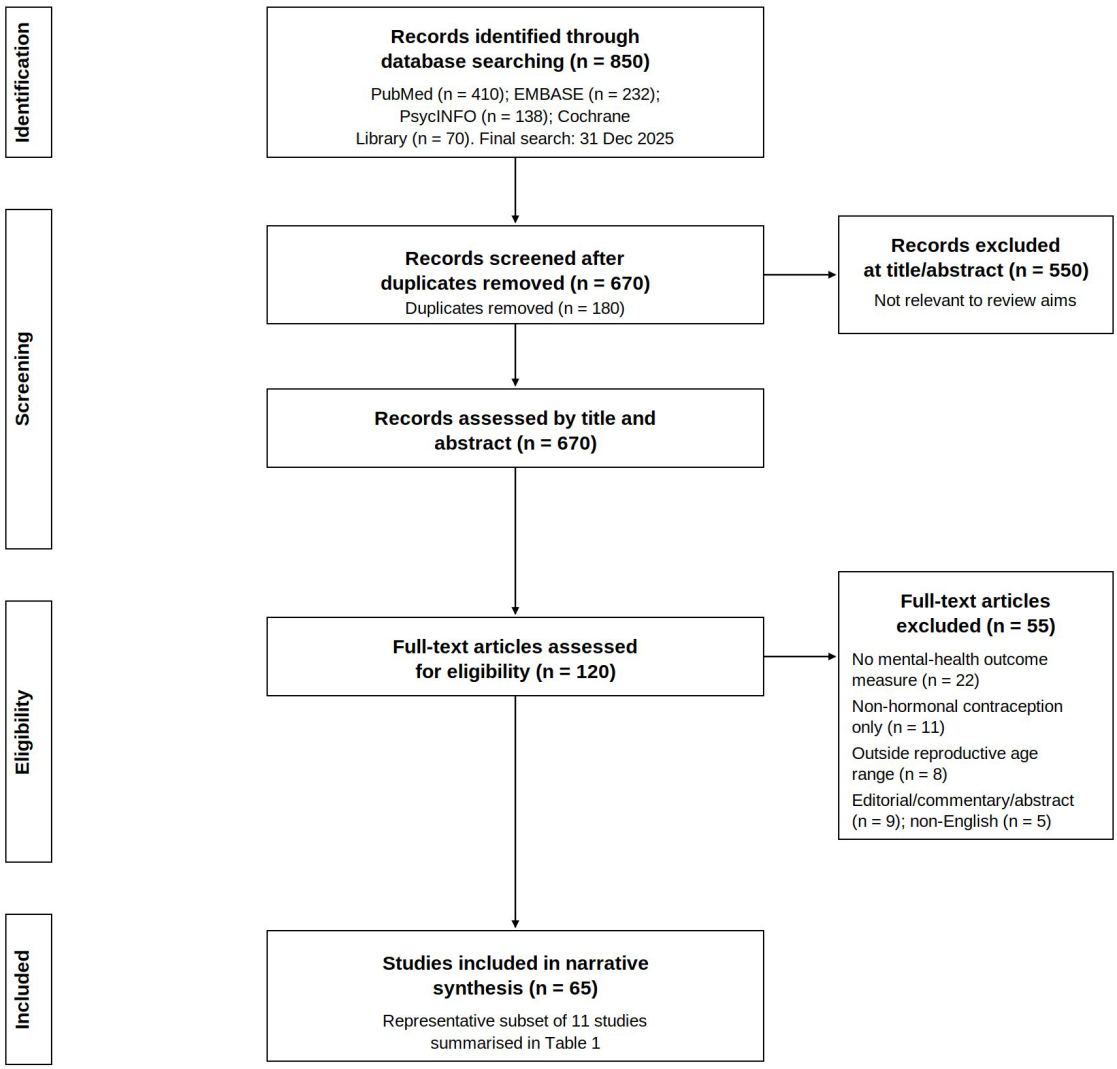
Flow diagram of study identification, screening, and selection. Flow of records through the narrative review, from database identification (n = 850) through duplicate removal and title/abstract screening to full-text assessment (n = 120) and final inclusion (n = 65). Counts reflect the authors’ record of the selection process and are presented to improve transparency rather than to denote a registered systematic-search audit trail.

The current literature presents a complex picture regarding the relationship between hormonal contraceptive use and mental health outcomes. While some studies suggest protective associations, particularly for anxiety and in certain populations, others indicate potential risks, especially for depression in vulnerable groups. It is important to state at the outset of this discussion that the evidence base is predominantly observational and heterogeneous, that its overall certainty is low to moderate, and that the associations described below should not be read as established causal effects (21). **Figure 2** presents a conceptual framework integrating the proposed biological mechanisms, moderating factors, mental health outcomes, and clinical implications identified in this review; it is intended as a hypothesis-generating map of the field rather than a depiction of confirmed causal pathways.

### 4.2 Individual risk assessment

#### 4.2.1 Patient-specific risk factors

Given the variable psychological responses to hormonal contraception, a personalized evaluation is important when selecting a contraceptive method, particularly among women at increased mental health risk. The association between HC use and mood may be influenced by age, especially during adolescence and young adulthood. Evidence from Doornweerd et al. (28) suggested that OC use in adolescents may help stabilize mood trajectories and may protect against anxiety and depression symptoms. However, this finding differs from studies such as Anderl, Li and Chen (27) and Skovlund et al. (26), which link adolescent HC use with an elevated risk of antidepressant use. These contrasting results indicate that the age of contraceptive initiation may influence emotional outcomes, but a cause-and-effect relationship cannot be established because of unmeasured factors including sexual behavior, relationship status, and psychological stress. Given these inconsistencies, adolescent patients should be carefully evaluated and their mood monitored over time.

A woman’s mental health history and previous experience with HCs may help predict future tolerance. Women who discontinued OCs because of mood effects were more likely to report isolation and mistrust, whereas women who did not experience adverse effects reported a lower risk of depression and lower scores on traits such as somatic anxiety and stress susceptibility (44). McCloskey et al. (30) noted that women with pre-existing psychiatric conditions using COCs experienced mood stabilization and a lower risk of suicide attempts. These conflicting findings may partly reflect selection and attrition effects: women who tolerate OCs are more likely to continue using them and to be captured as current users, while those who experience adverse effects discontinue early and drop out of prevalent-user samples, a pattern that can bias apparent associations in either direction (21). Clinicians should therefore consider past mental health history, prior HC use, and the reasons for any discontinuation as part of a comprehensive assessment.

#### 4.2.2 Method-specific considerations

The type, duration, dose, and formulation of HC may influence mental health outcomes. COCs are the most widely studied and have reported favorable outcomes; for example, low-dose monophasic COCs such as Minulet have been associated with reduced depression, anxiety, and mood lability, and anti-androgenic progestins such as drospirenone with fewer adverse mood effects and an indication for premenstrual dysphoric disorder (48). In contrast, progestin-only methods may carry a higher risk of mood-related effects, with one small pilot study reporting higher depression scores among progestin-only pill users than among combined-pill users or non-users (51); however, conflicting evidence suggests a protective association in some populations (30), creating genuine inconsistency in the risk-benefit picture. LARCs such as the levonorgestrel IUD provide long-term contraception with limited systemic exposure; some studies suggest improved quality of life and sexual functioning, whereas others associate LNG-IUD use with mood disturbance (31), and subtle effects or early removal can occur with extended use (52). Finally, Noachtar, Frokjaer and Pletzer (55) reported significant mood deterioration during the pill pause regardless of formulation, comparable to natural-cycle fluctuations, suggesting that continuous regimens may alleviate withdrawal-related symptoms, although de Wit et al. (42) found no fewer depressive symptoms with continuous versus cyclic ethinylestradiol/norgestimate.

### 4.3 Clinical implications for family physicians

Before presenting specific suggestions, we wish to be explicit about their evidentiary status. The screening questions, risk-stratification categories, and follow-up schedule proposed below are expert-informed, consensus-based clinical guidance derived from a low-to-moderate-certainty evidence base. They are not formal, evidence-based practice guidelines, and they have not been validated prospectively in primary care. They are offered to support, not to replace, individualized clinical judgment and shared decision-making.

#### 4.3.1 Pre-prescription screening

This review found that a substantial portion of the literature describes an association between hormonal contraceptive use and adverse mood outcomes in some users, and that certain groups, including women with pre-existing mental health conditions and those starting hormonal contraception during adolescence, may be more susceptible. Pre-prescription screening therefore serves two purposes: identifying women who may benefit from closer monitoring, and helping to guide selection among the available hormonal options. We suggest screening questions such as the following:

• “Have you ever been diagnosed with depression, anxiety, or other mental health conditions?”

• “Have you ever taken medication for mood or mental health concerns?”

• “Do you have a family history of mental health disorders?”

• “Have you noticed mood changes with previous hormonal contraceptives or during your menstrual cycle?”

• “Have you ever had thoughts of harming yourself?”

Validated instruments such as the Patient Health Questionnaire-9 (PHQ-9), Generalized Anxiety Disorder-7 (GAD-7), and Columbia-Suicide Severity Rating Scale (C-SSRS) can be used to detect depression, anxiety, and suicide risk. These recommended screening questions and validated instruments are summarized in **Table 2**.

**Table 2:** Pre-prescription mental health screening questions.

Recommended screening questions before initiating hormonal contraception:
“Have you ever been diagnosed with depression, anxiety, or other mental health conditions?” “Have you ever taken medication for mood or mental health concerns?” “Do you have a family history of mental health disorders?” “Have you noticed mood changes with previous hormonal contraceptives or during your menstrual cycle?” “Have you ever had thoughts of harming yourself?”
Validated screening instruments:
Patient Health Questionnaire-2 (PHQ-2): rapid initial depression screen Patient Health Questionnaire-9 (PHQ-9): depression severity assessment Generalized Anxiety Disorder-7 (GAD-7): anxiety severity assessment Columbia-Suicide Severity Rating Scale (C-SSRS): suicide risk evaluation

#### 4.3.2 Informed consent and counseling

Patients being prescribed hormonal contraception should be made aware of the potential risks and benefits of treatment. We recommend that physicians discuss these aspects thoroughly, set realistic expectations regarding outcomes and possible side effects, and obtain informed consent before initiation. Physicians should remain current with the relevant literature in order to provide balanced and accurate information.

#### 4.3.3 Monitoring and follow-up strategies

Mood-related and psychiatric side effects do not always appear abruptly; they can develop gradually and may take up to 6 months to peak (26). Monitoring of patients prescribed hormonal contraceptives may therefore help detect emerging difficulties and allow timely intervention. We suggest a follow-up schedule that includes a 3-month initial review for new users, a 6-month review if there are no concerns, an annual comprehensive review, and prompt contact for concerning symptoms. A detailed schedule with recommended actions and screening tools is provided in **Table 3**. We acknowledge, however, that structured psychiatric monitoring at fixed intervals may not be feasible in many primary care settings, where time, reimbursement, and administrative support are limited; surveys of primary care physicians show that even familiar tools such as the GAD-7 are used routinely by only a minority of clinicians (57). This schedule should therefore be regarded as a proposed approach to be adapted to local resources and individual risk, rather than as a fixed standard of care.

**Table 3:** Recommended follow-up schedule for mental health monitoring during hormonal contraceptive use.

Timepoint	Action	Tools/Focus
3 months (initial follow-up for all new users)	Assess mood changes, side effects, adherence, and satisfaction with method	PHQ-9, GAD-7; open-ended questions about mood, sleep, appetite, energy
6 months (if no concerns at 3-month visit)	Re-evaluate mental health status; compare to baseline screening scores	PHQ-9, GAD-7; discuss emerging symptoms; assess need for method adjustment
12 months (annual comprehensive review)	Comprehensive mental health and contraceptive review; reassess risk stratification	Full mental health screening; medication review; satisfaction; continuation vs. switch
As needed (immediate contact for concerning symptoms)	Urgent assessment for new or worsening mood symptoms, suicidal ideation, or functional impairment	C-SSRS if indicated; consider psychiatric referral; evaluate method change

For moderate- and high-risk patients, more frequent monitoring may be warranted, particularly during the first 6 months of use when mood-related symptoms are most likely to emerge (26). This schedule is a proposed approach to be adapted to the resources of each setting rather than a fixed standard of care.

### 4.4 Proposed clinical decision-making framework

**Figure 3:**
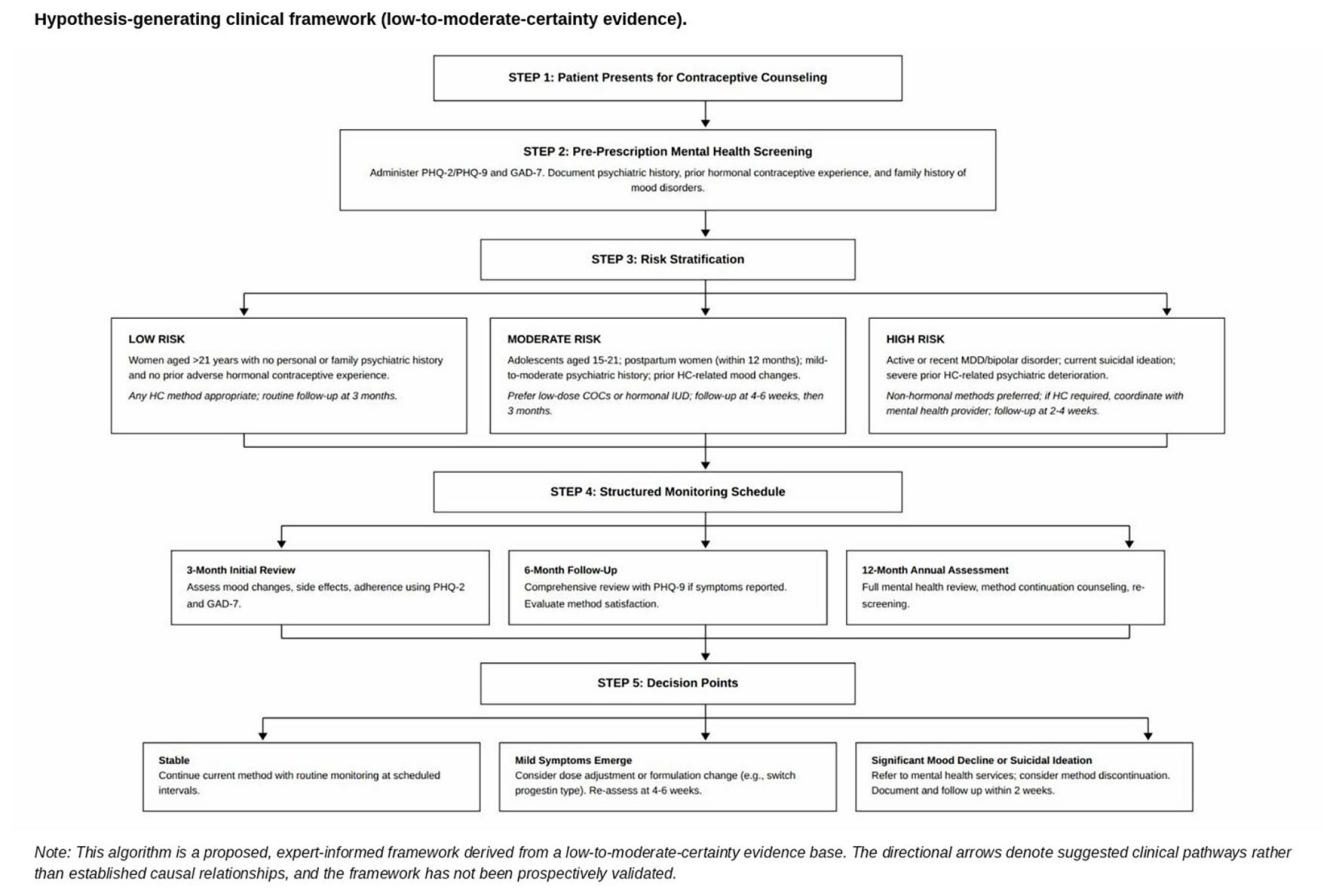
Proposed clinical decision-making algorithm for contraceptive counseling and mental health monitoring in family practice. The algorithm outlines pre-prescription screening, risk stratification (**Table 4**), an individualized monitoring schedule, and decision points for intervention. This figure is a proposed, hypothesis-generating clinical framework rather than a validated guideline; it reflects a low-to-moderate-certainty evidence base, and the directional arrows denote suggested clinical pathways rather than established causal relationships.

Based on the evidence reviewed, we propose a clinical decision-making framework for contraceptive counseling that incorporates mental health risk stratification (**Table 4**), method selection, structured monitoring, and decision points for intervention (**Figure 3**). Consistent with the low-to-moderate certainty of the underlying evidence, this algorithm is presented as a proposed, hypothesis-generating framework rather than a validated clinical guideline, and the directional arrows indicate suggested clinical pathways rather than established causal relationships.

#### 4.4.1 Low-risk patients

Low-risk patients can be identified as healthy women over the age of 21 with no pre-existing mental health conditions. Despite their lower risk profile, these patients still warrant standard counseling and monitoring when initiating contraception, given the potential for depression or anxiety documented in several studies; for example, one study found an increased risk of depression during the first two years of oral contraceptive use, underscoring the value of careful monitoring during this period (18). When selecting a method, each option presents both benefits and risks, and some findings remain contradictory or insufficiently researched. For low-risk patients, any hormonal method may be considered appropriate, provided routine medical follow-up and periodic mental health review are offered.

#### 4.4.2 Moderate-risk patients

Moderate-risk patients include those with factors that increase vulnerability to mood disturbance, such as postpartum women, adolescents, and women with a history of mild-to-moderate mental health conditions. For some postpartum patients, initiating hormonal contraception has been associated with an increased risk of depression, warranting caution and careful counseling (19). Adolescents experiencing hormonal and mood changes may be more likely to develop depression following OC initiation (18), and adolescents using contraceptives tend to have higher rates of antidepressant or psychotropic medication use, emphasizing the value of education, counseling, and frequent monitoring (26, 27). Given the contradictory nature of some data, providers should engage in shared decision-making and individualize choices. For moderate-risk patients, low-dose monophasic COCs, which have been associated with improvements in mood and reductions in depression and anxiety, may be a suitable option; non-hormonal alternatives may also be considered given the associations between hormonal contraceptives and mood disturbance in vulnerable groups (17). Where hormonal contraception is needed, hormonal IUDs may present a lower risk of mood-related effects owing to localized release, although the evidence is limited and sometimes conflicting (52); enhanced counseling and regular monitoring are advisable.

#### 4.4.3 High-risk patients

High-risk patients include those with a history of severe mental health disorders such as major depressive disorder or bipolar disorder, who may be particularly vulnerable to mood-related effects. A thorough, individualized risk-benefit discussion should precede initiation of any method, including review of psychiatric history and current symptoms, the potential hormonal impact on mood, and the patient’s goals and priorities. Because of the contradictory mood effects reported, non-hormonal options such as copper IUDs, condoms, or fertility awareness methods may be considered as first-line choices for high-risk patients, as these avoid systemic hormone exposure. If hormonal methods are chosen, they should be used cautiously, under close psychiatric supervision, with regular monitoring. Women with pre-existing psychiatric disorders may be more vulnerable to adverse mood effects and require closer monitoring; Skovlund et al. (26) reported elevated hazard ratios for antidepressant use in teenagers using hormonal contraception, mainly progestin-only pills, and Anderl, Li and Chen (27) noted that adolescent users were more likely to develop depression in adulthood. Conversely, McCloskey et al. (30) found that hormonal contraceptives had minimal or even positive effects in some women with psychiatric disorders. Because individuals respond differently, involving a mental health specialist may be appropriate, particularly for those with a history of mood instability or previous adverse reactions to hormonal contraception.

### 4.5 Addressing contraceptive myths and fears

Some patients express fear or hesitation before starting hormonal contraceptives because of concerns about depression, anxiety, or mood swings. This often reflects personal experience or social media misinformation, despite limited evidence for psychological harm in most users.

Our review provides a more balanced overall picture. Most included studies found no significant association between OCP use and depression or anxiety. For example, O’Connell, Davis and Kerns (58) found that depressive symptoms in adolescent OCP users were not significantly different from non-users. Other studies suggested protective or stabilizing effects of hormonal contraception on mood; Paoletti et al. (39) reported that women taking OCPs had fewer symptoms of anxiety and paranoia than naturally cycling women, and Kim et al. (40) suggested that hormonal contraceptive use may even reduce the long-term risk of Alzheimer’s disease in depressed women.

These fears are amplified by two related phenomena that deserve explicit clinical attention. The first is the nocebo effect, in which negative expectations generate or intensify symptoms. Placebo-controlled data are instructive here: a classic synthesis found that nonspecific symptoms such as mood change, headache, and reduced libido were reported almost as often by women taking placebo as by those taking active oral contraceptives, indicating that a substantial portion of attributed side effects is not pharmacological (59). Consistent with this, Robinson et al. (38) reported that women given placebo pills described emotional side effects as frequently as those given active hormones, and a recent survey found that women’s pre-existing beliefs about medicines predicted the side effects they subsequently experienced on the contraceptive pill (60).

The second phenomenon is the influence of social media and online discourse. A 2025 content analysis of the most-viewed contraception videos on TikTok, which together attracted billions of views, found that only a small minority were produced by medical professionals, that negative personal testimony predominated, and that content frequently promoted a shift toward “natural” methods alongside growing distrust of clinicians (61). Related analyses have documented how such platforms shape contraceptive decision-making and amplify side-effect narratives, including mood-related concerns, often without clinical context (62, 63). Family physicians should therefore anticipate that patients may arrive already influenced by these narratives, and should be prepared to engage with them respectfully rather than dismissively.

Clinicians must responsibly balance respect for patient autonomy with evidence-based counseling. While it is important to understand patients’ concerns and previous negative experiences, it is equally essential to provide accurate, up-to-date information so that decisions are guided by evidence rather than fear. Reassurance grounded in the actual evidence, rather than blanket statements, tends to be more credible to patients and more durable when they encounter contrary anecdotes online.

### 4.6 Integration with mental health care

Referral to a mental health professional should be considered when a patient has a pre-existing psychiatric condition that is not well controlled, develops new or worsening symptoms after initiating hormonal contraception, has a history of mood-related side effects from previous contraceptive use, or expresses suicidal thoughts or functional impairment related to mood symptoms.

In our review, while most studies showed that women tolerate contraception well, a subset, mainly adolescents and those with psychiatric comorbidities, may benefit from early psychiatric evaluation to ensure safe contraceptive use. Combining reproductive and mental health services can significantly improve outcomes, ensuring that contraceptive decisions are made with emotional well-being in mind. This is especially important for high-risk patients who may need ongoing evaluation and adjustments in therapy over time.

When reproductive health and psychiatric care are coordinated, the practical result is fewer unnecessary contraceptive discontinuations and earlier identification of genuine mood deterioration. This also empowers patients to remain on contraception safely without unnecessary discontinuation based on unclarified symptoms or assumptions. The key messages for clinical practice arising from this review are summarized in **Table 5**.

**Table 4:** Risk stratification.

Risk level	Patient profile	Recommended approach
LOW RISK	Healthy women aged >21 years; no personal or family psychiatric history; no prior adverse HC experience	Any HC method appropriate; standard counseling; routine follow-up at 3 months
MODERATE RISK	Adolescents aged 15-21; postpartum women (within 12 months); mild-to-moderate psychiatric history; prior HC-related mood changes	Prefer low-dose COCs with anti-androgenic progestins (e.g., drospirenone) or hormonal IUDs; enhanced counseling on mood monitoring; follow-up at 4-6 weeks, then 3 months
HIGH RISK	Active or recent MDD/bipolar disorder; current suicidal ideation; severe prior HC-related psychiatric deterioration; current psychotropic medication	Prefer non-hormonal methods (copper IUD, barrier methods); if HC required, use under close psychiatric supervision; coordinate with mental health provider; follow-up at 2-4 weeks

**Table 5:** Key messages for clinical practice.

Hormonal contraceptives are well tolerated by most women, but certain subgroups warrant greater vigilance, including adolescents, postpartum women, and those with pre-existing psychiatric conditions. Pre-prescription screening using validated tools (PHQ-9, GAD-7) can help identify women who may benefit from closer monitoring before initiating hormonal contraception. Progestin-only methods and contraceptive initiation during adolescence have been more consistently associated with mood-related risks, while certain combined oral contraceptives (particularly those with anti-androgenic progestins) may be associated with mood stabilization. Shared decision-making should guide every clinical encounter; method selection should account for individual biological, psychological, and social factors rather than a one-size-fits-all approach. Structured follow-up (at 3 months, 6 months, and annually) with re-administration of screening tools can help detect gradual-onset mood changes, which may take up to 6 months to peak. Integration of reproductive and mental health services may improve outcomes; referral to a mental health specialist should be considered for patients with pre-existing psychiatric conditions, new or worsening mood symptoms, or suicidal ideation. Balanced, evidence-based counseling should address both the benefits and potential mood-related risks of hormonal contraception, while addressing common myths amplified by social media.

### 4.7 Future research directions

Consistent with the need to prioritize a manageable set of questions, we highlight three research gaps as most important. First, longitudinal studies that follow adolescents from before initiation, through active use, and into the post-discontinuation period are needed to clarify the timing, persistence, and reversibility of any mood effects. Second, the field requires standardized, validated psychiatric outcome measures applied consistently across studies, so that findings become comparable and pooling becomes meaningful. Third, formulation-specific analyses, distinguishing progestin type, dose, and route of administration, are needed to move beyond treating hormonal contraception as a single, undifferentiated exposure. The directions that follow elaborate on these three priorities.

Future research should employ longitudinal designs tracking individuals over time to adequately characterize the long-term psychological correlates of HC use, particularly when initiated during adolescence. Ideally, such studies should begin before HC initiation, continue through active use, and extend into the post-discontinuation stage to assess possible delayed or residual effects.

Beyond broad dichotomous comparisons (e.g., adolescence vs. adulthood), researchers should incorporate pubertal growth and developmental milestones (64, 65). Neurodevelopmental changes affecting stress reactivity and emotional regulation occur at various points in adolescence and may alter the sensitivity of the brain to exogenous hormones (10). Studies of phase-dependent effects of COCs should also be included, as differences in memory, stress response, and brain connectivity have been reported between active and inactive pill phases (66–69).

#### Incorporating lifetime HC use and attrition data

Research such as Morssinkhof et al. (70) indicates that within-person designs are needed to address inter-individual variability in susceptibility to mood effects. Attrition driven by adverse mood outcomes can create sampling bias in which prevalent-user samples are composed disproportionately of healthy users, thereby underestimating the true effect of HC on mood (26). A key recommendation is that studies report the lifetime history of HC use, including age of initiation, duration of use, and reasons for discontinuation (71).

#### Moving towards individualized medicine and contraceptive matching

Future research should incorporate a personalized-medicine model by establishing psychological, hormonal, and genetic markers of risk for mood disturbance with HC use (72). Tailoring contraceptive options to these individual differences may improve adherence and mood outcomes (42). Efforts are underway to develop new progestins with minimized mood-altering effects and to investigate local hormone-delivery devices, such as hormonal IUDs and patches, that may reduce systemic exposure (73). Early identification and management of mood symptoms may be facilitated by digital health platforms and telemedicine solutions that enable real-time symptom and mood tracking (73). Such platforms could also support the assembly of larger, prospectively collected datasets to inform more individualized contraceptive counseling.

#### Biomarker identification and neurobiological mechanisms

Pursuing biological predictors of susceptibility to mood change with HC use is an important area for further research; candidates include inflammatory markers, urinary or salivary hormone profiles, and genetic polymorphisms associated with neuropsychiatric risk (13). Cortisol profiles, which have been found to be reduced or increased in HC users, are additional candidates related to stress sensitivity (74). Neuroimaging with positron emission tomography (PET) could reveal how hormonal contraceptives affect glucocorticoid and sex-hormone receptors in the brain (75), and resting-state connectivity and stress-activated neuronal activity across contraceptive phases could further illuminate the neurobiological basis of HC-related emotional and cognitive changes (69).

In addition to biological factors, future research should systematically investigate psychosocial moderators of HC-related mental health outcomes, such as personality dimensions (e.g., risk-taking, trait anxiety), relationship functioning, sexual behavior, education level, religiosity, and beliefs about medication (76, 77). These factors may influence both HC initiation and the psychological response to it, introducing selection bias. Comparative studies in nonhuman primates may also help isolate the psycho-behavioral effects of exogenous hormones under experimental conditions (78–81).

### 4.8 Limitations of current evidence

#### Study design limitations

The main issue with the available literature is its heavy reliance on observational designs, including cross-sectional, retrospective cohort, and case-control studies (16). These designs are useful for identifying associations between hormonal contraceptives and psychological outcomes but limit the ability to infer causation. Many studies measured outcomes at a single time point, preventing proper assessment of the temporal relationship between symptom onset and HC initiation (18). Included prospective studies often lacked sufficient follow-up to determine long-term outcomes, and randomized controlled trials, the gold standard for causal inference, were scarce. Inconsistent outcome definitions, particularly in how depression and anxiety were measured, further limit comparability; some studies used standardized diagnostic tools (28) while others relied on self-report (35). Because symptomatic users may discontinue or switch methods, reverse causation is also possible, whereby mood influences contraceptive behavior rather than the reverse. Methodologists have argued that overcoming these obstacles will require new-user (incident-user) designs and target-trial emulation that align the analytic start time with treatment initiation (21, 82).

#### Selection bias issues

Selection bias is a significant limitation of the current literature. Many included studies used convenience sampling or recruited from specific subpopulations such as university students, clinic patients, or online survey responders (83), which may not represent the broader population of HC users, particularly with respect to baseline mental health or healthcare access. Some studies had high dropout rates (84) or strict exclusion criteria that removed women with pre-existing psychological disorders, yielding findings from healthier subgroups (85). Two specific mechanisms deserve emphasis. The healthy-user effect arises because women who tolerate hormonal contraception tend to continue using it and are therefore over-represented among current users, while those who experience adverse mood effects discontinue and are reclassified as past users or excluded; cross-sectional comparisons of current users can consequently appear falsely reassuring (21, 47). The related problem of attrition bias, or depletion of susceptibles, means that early discontinuation by the most susceptible women can lead prevalent-user cohorts to underestimate the true incidence of mood effects (20, 26). Together these effects can bias results in either direction and are a central reason why current-user and never-user comparisons must be interpreted cautiously.

#### Confounding factors

Confounding is a critical issue in mental health research. While many studies adjusted for variables such as age, socioeconomic status, and educational attainment, others did not (21). Important confounders include prior history of mood disorders, stressful life events, coexisting medical conditions, substance use, and concurrent use of medications such as antidepressants or mood stabilizers. When these variables are not adequately addressed, it becomes difficult to determine whether observed associations reflect an effect of hormonal contraception or underlying differences between users and non-users. Confounding by indication is particularly relevant here, because women are frequently prescribed hormonal contraception for non-contraceptive reasons, including premenstrual dysphoric disorder, acne, and menstrual disorders, conditions that are themselves associated with mood symptoms; such women may differ systematically from those using contraception solely to prevent pregnancy, biasing comparisons unless indication is accounted for (17).

#### Generalizability concerns

Finally, generalizability is limited because most studies were conducted in high-income, Western countries, constraining applicability to women in different cultural, economic, and healthcare contexts. Many participants were relatively young and well-educated, often recruited from universities or online forums (83), resulting in under-representation of women from rural areas, those of lower socioeconomic status, and those with limited healthcare access. Cultural and socioeconomic variability may shape both the experience and the reporting of mood symptoms, and the findings summarized here should therefore not be assumed to be universally generalizable. These observations highlight the need for more inclusive and representative research.

Taken together, the predominantly observational nature of the evidence, the susceptibility to selection and attrition bias, the potential for confounding by indication and reverse causation, and the limited generalizability of existing samples are the reasons the certainty of the overall evidence base is low to moderate, and the reason the clinical recommendations in this review are presented as proposed, expert-informed guidance rather than as definitive practice standards.

## 5 Conclusion

The relationship between hormonal contraceptives and mental health is complex and highly individual. While most women can use hormonal contraceptives without experiencing adverse psychological symptoms, certain groups appear more vulnerable. Women with a prior history of depression or anxiety, adolescents, and those with additional psychological risk factors may be at higher risk of developing or worsening symptoms, although, as discussed above, the observational evidence underlying these patterns cannot establish causation. This underscores the value of thorough assessment before contraceptive initiation and attentive monitoring during use.

Screening tools for depression and anxiety can be integrated into clinical visits to help physicians recognize potentially susceptible patients before initiating hormonal contraceptives. Once contraceptives are prescribed, follow-up can help ensure safety and effectiveness while identifying adverse psychological symptoms that may prompt review of the chosen method in partnership with the patient.

Contraceptive counseling works best when it treats the woman in front of the clinician as a whole person rather than a risk category. Biological vulnerability, psychological history, and social circumstances all bear on which method is likely to be tolerated well and maintained over the long term.

Honest, evidence-grounded counseling means neither dismissing patient concerns nor amplifying them beyond what the data support. Monitoring at structured intervals, with validated screening tools re-administered at each visit, can help clinicians detect gradual mood changes before they become clinically significant. Women who know what to watch for, and who know that their clinician is watching too, may be more likely to report early symptoms than to quietly discontinue an otherwise effective contraceptive.

The practical direction from this body of evidence is straightforward even though the underlying science is still evolving: screen systematically before prescribing, match the method to the individual rather than applying a single approach across all patients, and treat mood as a clinical outcome deserving structured follow-up. These suggestions are offered as proposed, expert-informed guidance, and their intensity should be adapted to the resources of each primary care setting and the risk profile of each patient.

In primary care, where most contraceptive decisions are made, integrating routine psychiatric enquiry into contraceptive visits is both practical and valuable. Family physicians hold longitudinal knowledge of their patients that no single specialist visit can replicate. That position carries a corresponding responsibility to keep current with an evidence base that is still developing, and to communicate uncertainty honestly rather than defaulting to either blanket reassurance or unnecessary restriction.

## Glossary

BDI: Beck Depression Inventory
C-SSRS: Columbia Suicide Severity Rating Scale
CIDI-SF: Composite International Diagnostic Interview Short Form
COC: combined oral contraceptive
EE: ethinylestradiol
GABA: gamma-aminobutyric acid
GAD: generalized anxiety disorder
GAD-7: Generalized Anxiety Disorder 7-item Scale
HC: hormonal contraception
HPA: hypothalamic-pituitary-adrenal
IUD: intrauterine device
LARC: long-acting reversible contraceptive
LNG: levonorgestrel
LNG-IUD: levonorgestrel-releasing intrauterine device
mCPR: modern contraceptive prevalence rate
MDD: major depressive disorder
NHANES: National Health and Nutrition Examination Survey
OC: oral contraceptive
OCP: oral contraceptive pill
PD: panic disorder
PHQ-2: Patient Health Questionnaire 2-item
PHQ-9: Patient Health Questionnaire 9-item
PMDD: premenstrual dysphoric disorder
PMS: premenstrual syndrome
POP: progestin-only pill
PPD: postpartum depression
SANRA: Scale for the Assessment of Narrative Review Articles
